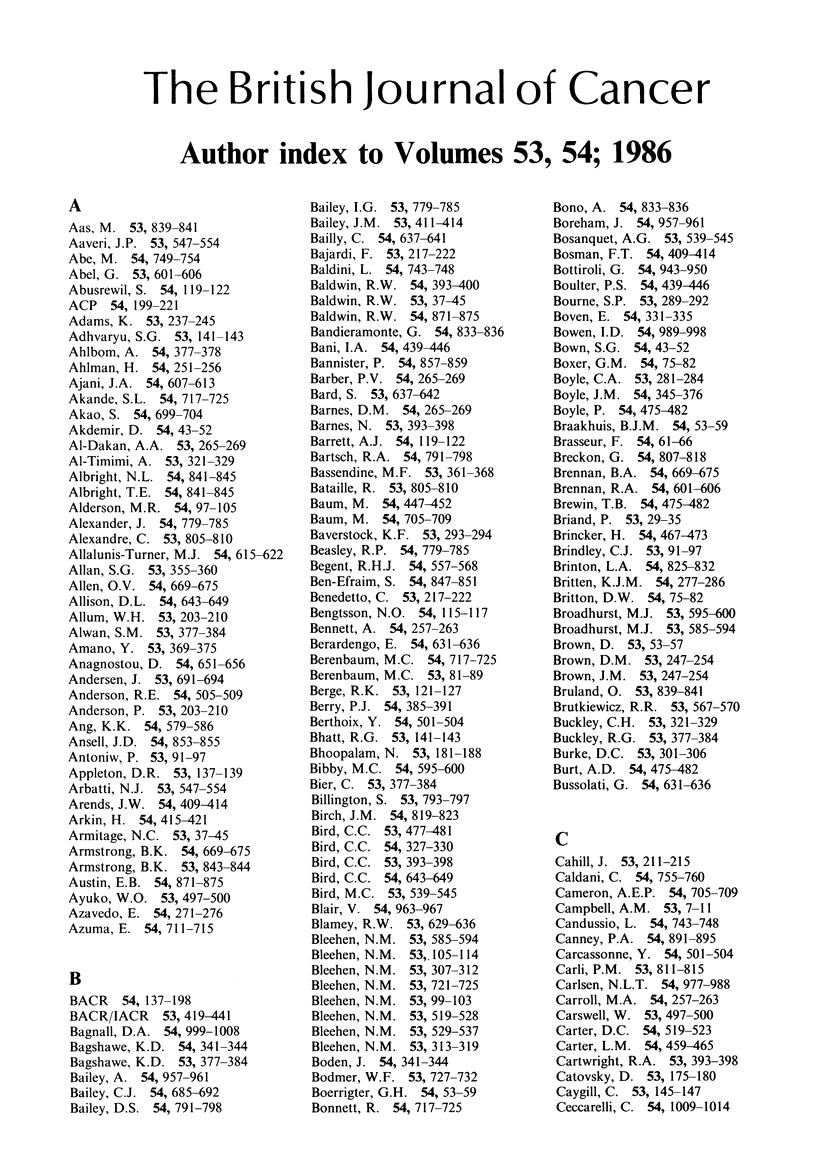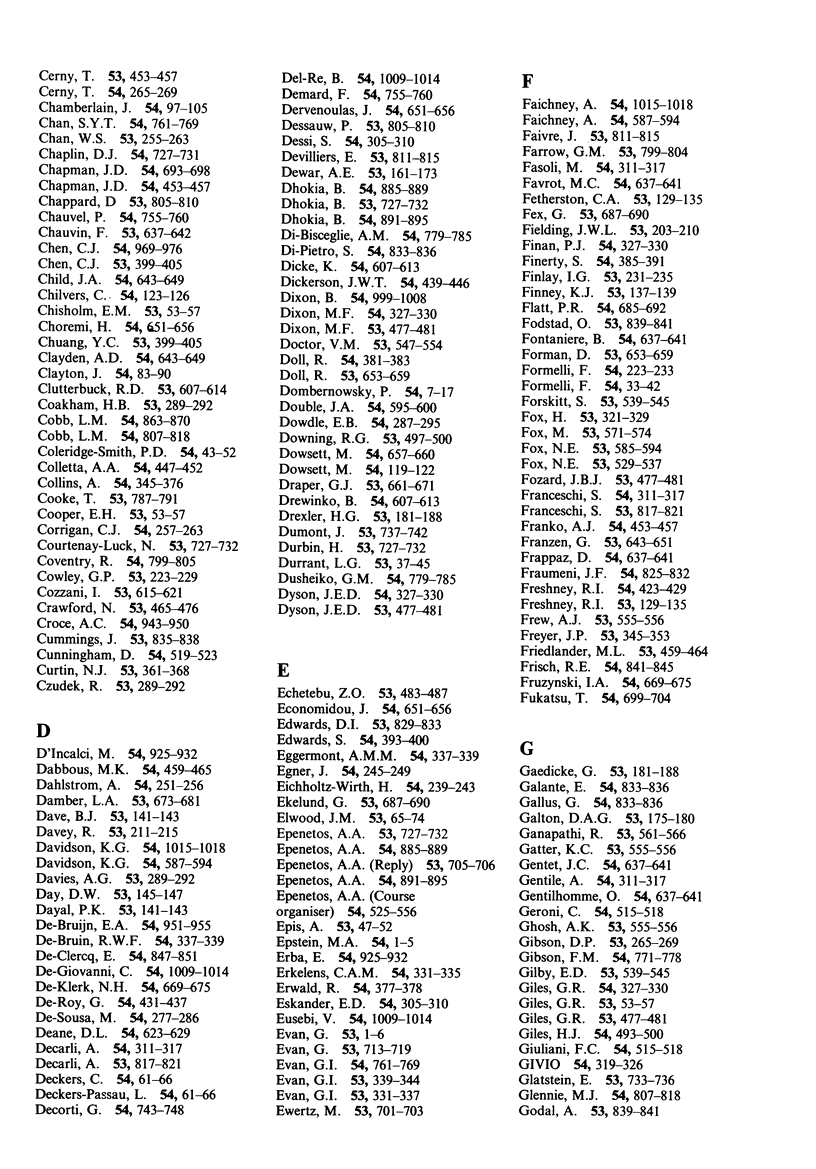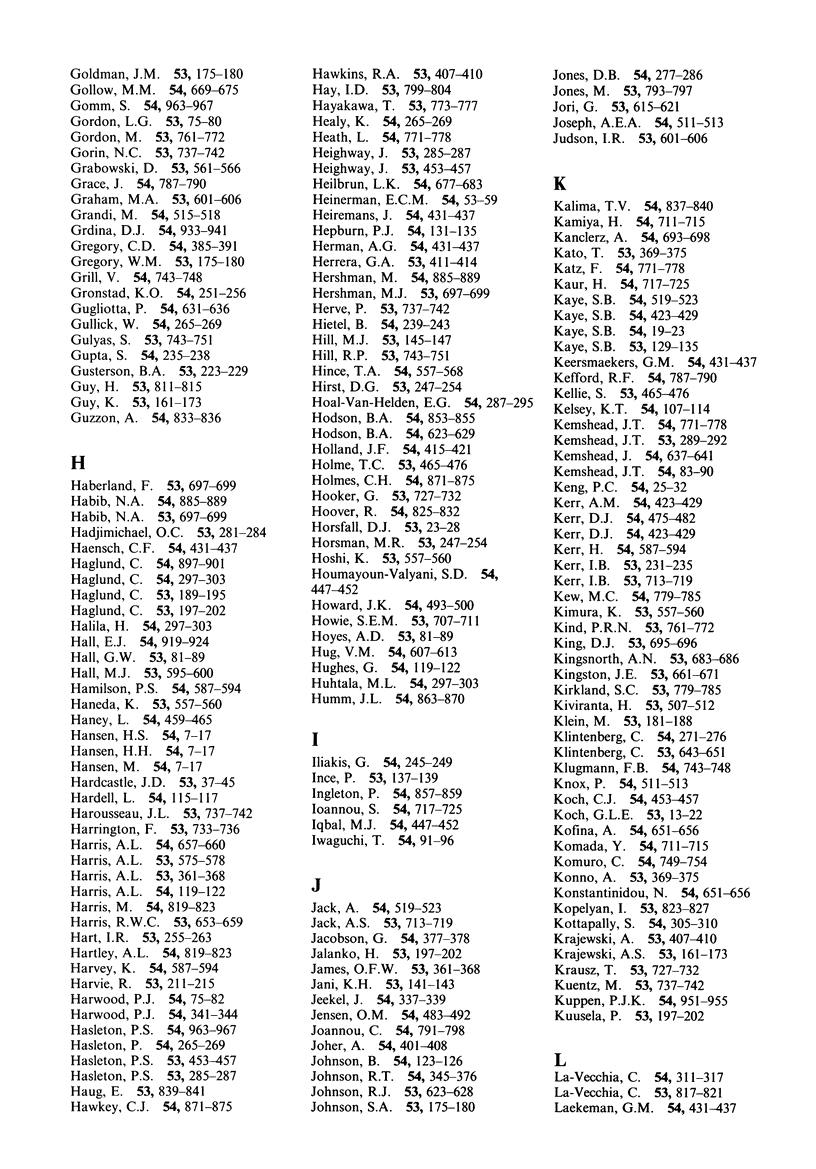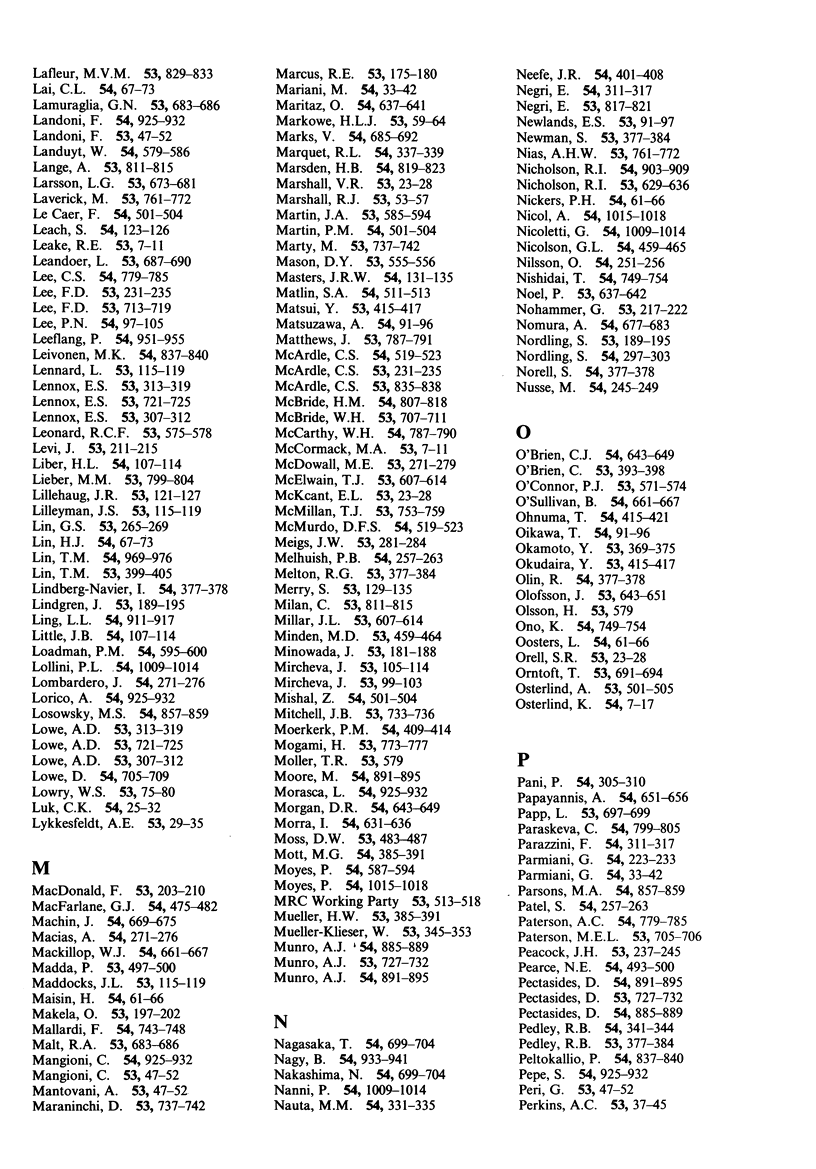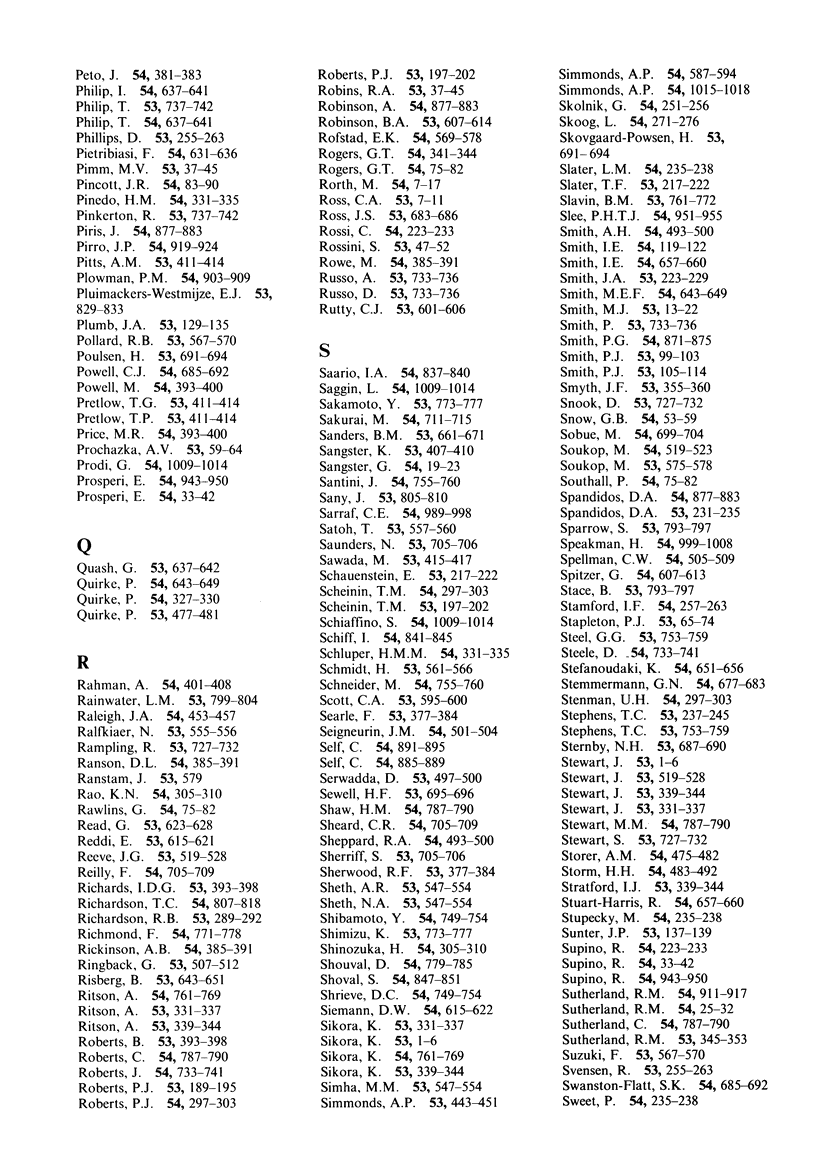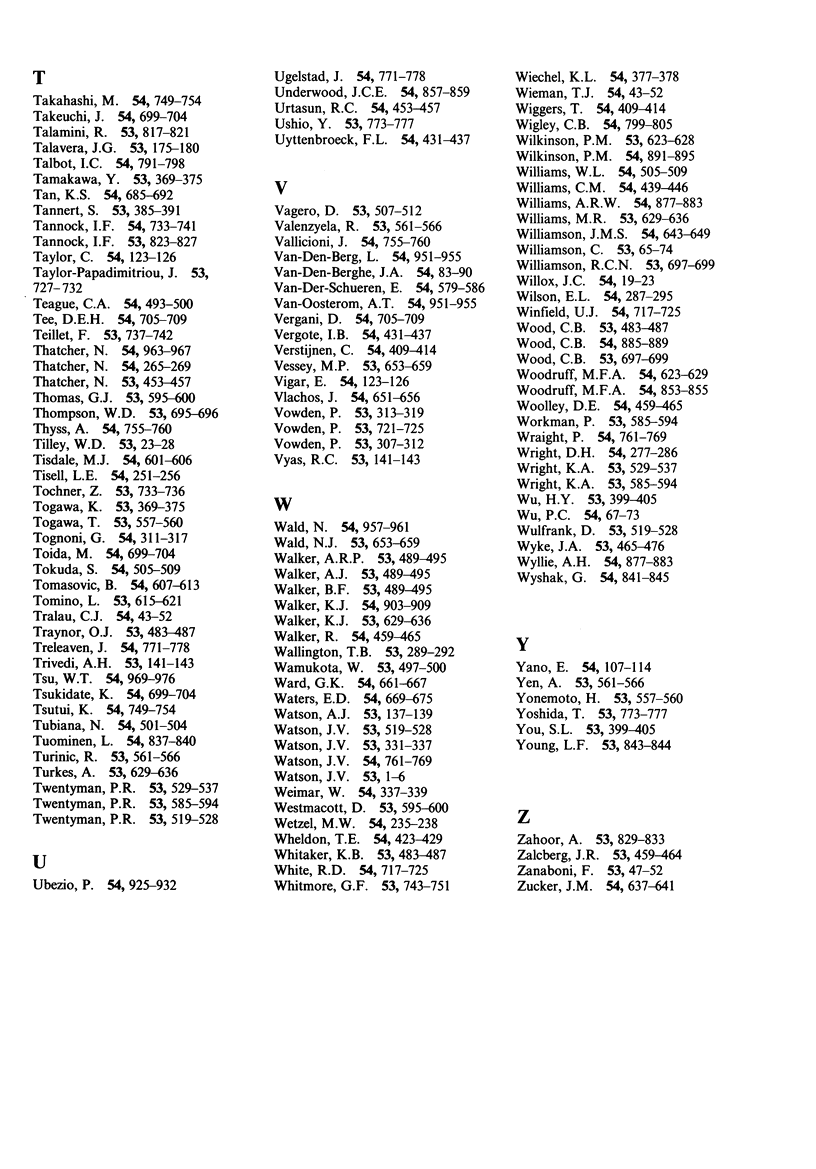# Author index to Volumes 53, 54; 1986

**Published:** 1986

**Authors:** 


### The British Journal of Cancer

### Author index to Volumes 53, 54; 1986

### A

Aas, M. **53,** 839–841
Aaveri, J.P. **53,** 547–554
Abe, M. **54,** 749–754
Abel, G. **53,** 601–606
Abusrewil, S. **54,** 119–122
ACP **54,** 199–221
Adams, K. **53,** 237–245
Adhvaryu, S.G. **53,** 141–143
Ahlbom, A. **54,** 377–378
Ahlman, H. **54,** 251–256
Ajani, J.A. **54,** 607–613
Akande, S.L. **54,** 717–725
Akao, S. **54,** 699–704
Akdemir, D. **54,** 43–52
Al-Dakan, A.A. **53,** 265–269
Al-Timimi, A. **53,** 321–329
Albright, N.L. **54,** 841–845
Albright, T.E. **54,** 841–845
Alderson, M.R. **54,** 97–105
Alexander, J. **54,** 779–785
Alexandre, C. **53,** 805–810
Allalunis-Turner, M.J. **54,** 615–622
Allan, S.G. **53,** 355–360
Allen, O.V. **54,** 669–675
Allison, D.L. **54,** 643–649
Allum, W.H. **53,** 203–210
Alwan, S.M. **53,** 377–384
Amano, Y. **53,** 369–375
Anagnostou, D. **54,** 651–656
Andersen, J. **53,** 691–694
Anderson, R.E. **54,** 505–509
Anderson, P. **53,** 203–210
Ang, K.K. **54,** 579–586
Ansell, J.D. **54,** 853–855
Antoniw, P. **53,** 91–97
Appleton, D.R. **53,** 137–139
Arbatti, N.J. **53,** 547–554
Arends, J.W. **54,** 409–414
Arkin, H. **54,** 415–421
Armitage, N.C. **53,** 37–45
Armstrong, B.K. **54,** 669–675
Armstrong, B.K. **53,** 843–844
Austin, E.B. **54,** 871–875
Ayuko, W.O. **53,** 497–500
Azavedo, E. **54,** 271–276
Azuma, E. **54,** 711–715

### B

BACR **54,** 137–198
BACR/IACR **53,** 419–441
Bagnall, D.A. **54,** 999–1008
Bagshawe, K.D. **54,** 341–344
Bagshawe, K.D. **53,** 377–384
Bailey, A. **54,** 957–961
Bailey, C.J. **54,** 685–692
Bailey, D.S. **54,** 791–798
Bailey, I.G. **53,** 779–785
Bailey, J.M. **53,** 411–414
Bailly, C. **54,** 637–641
Bajardi, F. **53,** 217–222
Baldini, L. **54,** 743–748
Baldwin, R.W. **54,** 393–400
Baldwin, R.W. **53,** 37–45
Baldwin, R.W. **54,** 871–875
Bandieramonte, G. **54,** 833–836
Bani, I.A. **54,** 439–446
Bannister, P. **54,** 857–859
Barber, P.V. **54,** 265–269
Bard, S. **53,** 637–642
Barnes, D.M. **54,** 265–269
Barnes, N. **53,** 393–398
Barrett, A.J. **54,** 119–122
Bartsch, R.A. **54,** 791–798
Bassendine, M.F. **53,** 361–368
Bataille, R. **53,** 805–810
Baum, M. **54,** 447–452
Baum, M. **54,** 705–709
Baverstock, K.F. **53,** 293–294
Beasley, R.P. **54,** 779–785
Begent, R.H.J. **54,** 557–568
Ben-Efraim, S. **54,** 847–851
Benedetto, C. **53,** 217–222
Bengtsson, N.O. **54,** 115–117
Bennett, A. **54,** 257–263
Berardengo, E. **54,** 631–636
Berenbaum, M.C. **54,** 717–725
Berenbaum, M.C. **53,** 81–89
Berge, R.K. **53,** 121–127
Berry, P.J. **54,** 385–391
Berthoix, Y. **54,** 501–504
Bhatt, R.G. **53,** 141–143
Bhoopalam, N. **53,** 181–188
Bibby, M.C. **54,** 595–600
Bier, C. **53,** 377–384
Billington, S. **53,** 793–797
Birch, J.M. **54,** 819–823
Bird, C.C. **53,** 477–481
Bird, C.C. **54,** 327–330
Bird, C.C. **53,** 393–398
Bird, C.C. **54,** 643–649
Bird, M.C. **53,** 539–545
Blair, V. **54,** 963–967
Blamey, R.W. **53,** 629–636
Bleehen, N.M. **53,** 585–594
Bleehen, N.M. **53,**. 105–114
Bleehen, N.M. **53,** 307–312
Bleehen, N.M. **53,** 721–725
Bleehen, N.M. **53,** 99–103
Bleehen, N.M. **53,** 519–528
Bleehen, N.M. **53,** 529–537
Bleehen, N.M. **53,** 313–319
Boden, J. **54,** 341–344
Bodmer, W.F. **53,** 727–732
Boerrigter, G.H. **54,** 53–59
Bonnett, R. **54,** 717–725
Bono, A. **54,** 833–836
Boreham, J. **54,** 957–961
Bosanquet, A.G. **53,** 539–545
Bosman, F.T. **54,** 409–414
Bottiroli, G. **54,** 943–950
Boulter, P.S. **54,** 439–446
Bourne, S.P. **53,** 289–292
Boven, E. **54,** 331–335
Bowen, I.D. **54,** 989–998
Bown, S.G. **54,** 43–52
Boxer, G.M. **54,** 75–82
Boyle, C.A. **53,** 281–284
Boyle, J.M. **54,** 345–376
Boyle, P. **54,** 475–482
Braakhuis, B.J.M. **54,** 53–59
Brasseur, F. **54,** 61–66
Breckon, G. **54,** 807–818
Brennan, B.A. **54,** 669–675
Brennan, R.A. **54,** 601–606
Brewin, T.B. **54,** 475–482
Briand, P. **53,** 29–35
Brincker, H. **54,** 467–473
Brindley, C.J. **53,** 91–97
Brinton, L.A. **54,** 825–832
Britten, K.J.M. **54,** 277–286
Britton, D.W. **54,** 75–82
Broadhurst, M.J. **53,** 595–600
Broadhurst, M.J. **53,** 585–594
Brown, D. **53,** 53–57
Brown, D.M. **53,** 247–254
Brown, J.M. **53,** 247–254
Bruland, O. **53,** 839–841
Brutkiewicz, R.R. **53,** 567–570
Buckley, C.H. **53,** 321–329
Buckley, R.G. **53,** 377–384
Burke, D.C. **53,** 301–306
Burt, A.D. **54,** 475–482
Bussolati, G. **54,** 631–636

### C

Cahill, J. **53,** 211–215
Caldani, C. **54,** 755–760
Cameron, A.E.P. **54,** 705–709
Campbell, A.M. **53,** 7–11
Candussio, L. **54,** 743–748
Canney, P.A. **54,** 891–895
Carcassonne, Y. **54,** 501–504
Carli, P.M. **53,** 811–815
Carlsen, N.L.T. **54,** 977–988
Carroll, M.A. **54,** 257–263
Carswell, W. **53,** 497–500
Carter, D.C. **54,** 519–523
Carter, L.M. **54,** 459–465
Cartwright, R.A. **53,** 393–398
Catovsky, D. **53,** 175–180
Caygill, C. **53,** 145–147
Ceccarelli, C. **54,** 1009–1014

Cerny, T. **53,** 453–457
Cerny, T. **54,** 265–269
Chamberlain, J. **54,** 97–105
Chan, S.Y.T. **54,** 761–769
Chan, W.S. **53,** 255–263
Chaplin, D.J. **54,** 727–731
Chapman, J.D. **54,** 693–698
Chapman, J.D. **54,** 453–457
Chappard, D **53,** 805–810
Chauvel, P. **54,** 755–760
Chauvin, F. **53,** 637–642
Chen, C.J. **54,** 969–976
Chen, C.J. **53,** 399–405
Child, J.A. **54,** 643–649
Chilvers, C. **54,** 123–126
Chisholm, E.M. **53,** 53–57
Choremi, H. **54,** 651–656
Chuang, Y.C. **53,** 399–405
Clayden, A.D. **54,** 643–649
Clayton, J. **54,** 83–90
Clutterbuck, R.D. **53,** 607–614
Coakham, H.B. **53,** 289–292
Cobb, L.M. **54,** 863–870
Cobb, L.M. **54,** 807–818
Coleridge-Smith, P.D. **54,** 43–52
Colletta, A.A. **54,** 447–452
Collins, A. **54,** 345–376
Cooke, T. **53,** 787–791
Cooper, E.H. **53,** 53–57
Corrigan, C.J. **54,** 257–263
Courtenay-Luck, N. **53,** 727–732
Coventry, R. **54,** 799–805
Cowley, G.P. **53,** 223–229
Cozzani, I. **53,** 615–621
Crawford, N. **53,** 465–476
Croce, A.C. **54,** 943–950
Cummings, J. **53,** 835–838
Cunningham, D. **54,** 519–523
Curtin, N.J. **53,** 361–368
Czudek, R. **53,** 289–292

### D

D'Incalci, M. **54,** 925–932
Dabbous, M.K. **54,** 459–465
Dahlstrom, A. **54,** 251–256
Damber, L.A. **53,** 673–681
Dave, B.J. **53,** 141–143
Davey, R. **53,** 211–215
Davidson, K.G. **54,** 1015–1018
Davidson, K.G. **54,** 587–594
Davies, A.G. **53,** 289–292
Day, D.W. **53,** 145–147
Dayal, P.K. **53,** 141–143
De-Bruijn, E.A. **54,** 951–955
De-Bruin, R.W.F. **54,** 337–339
De-Clercq, E. **54,** 847–851
De-Giovanni, C. **54,** 1009–1014
De-Klerk, N.H. **54,** 669–675
De-Roy, G. **54,** 431–437
De-Sousa, M. **54,** 277–286
Deane, D.L. **54,** 623–629
Decarli, A. **54,** 311–317
Decarli, A. **53,** 817–821
Deckers, C. **54,** 61–66
Deckers-Passau, L. **54,** 61–66
Decorti, G. **54,** 743–748
Del-Re, B. **54,** 1009–1014
Demard, F. **54,** 755–760
Dervenoulas, J. **54,** 651–656
Dessauw, P. **53,** 805–810
Dessi, S. **54,** 305–310
Devilliers, E. **53,** 811–815
Dewar, A.E. **53,** 161–173
Dhokia, B. **54,** 885–889
Dhokia, B. **53,** 727–732
Dhokia, B. **54,** 891–895
Di-Bisceglie, A.M. **54,** 779–785
Di-Pietro, S. **54,** 833–836
Dicke, K. **54,** 607–613
Dickerson, J.W.T. **54,** 439–446
Dixon, B. **54,** 999–1008
Dixon, M.F. **54,** 327–330
Dixon, M.F. **53,** 477–481
Doctor, V.M. **53,** 547–554
Doll, R. **54,** 381–383
Doll, R. **53,** 653–659
Dombernowsky, P. **54,** 7–17
Double, J.A. **54,** 595–600
Dowdle, E.B. **54,** 287–295
Downing, R.G. **53,** 497–500
Dowsett, M. **54,** 657–660
Dowsett, M. **54,** 119–122
Draper, G.J. **53,** 661–671
Drewinko, B. **54,** 607–613
Drexler, H.G. **53,** 181–188
Dumont, J. **53,** 737–742
Durbin, H. **53,** 727–732
Durrant, L.G. **53,** 37–45
Dusheiko, G.M. **54,** 779–785
Dyson, J.E.D. **54,** 327–330
Dyson, J.E.D. **53,** 477–481

### E

Echetebu, Z.O. **53,** 483–487
Economidou, J. **54,** 651–656
Edwards, D.I. **53,** 829–833
Edwards, S. **54,** 393–400
Eggermont, A.M.M. **54,** 337–339
Egner, J. **54,** 245–249
Eichholtz-Wirth, H. **54,** 239–243
Ekelund, G. **53,** 687–690
Elwood, J.M. **53,** 65–74
Epenetos, A.A. **53,** 727–732
Epenetos, A.A. **54,** 885–889
Epenetos, A.A. (Reply) **53,** 705–706
Epenetos, A.A. **54,** 891–895
Epenetos, A.A. (Course organiser) **54,** 525–556
Epis, A. **53,** 47–52
Epstein, M.A. **54,** 1–5
Erba, E. **54,** 925–932
Erkelens, C.A.M. **54,** 331–335
Erwald, R. **54,** 377–378
Eskander, E.D. **54,** 305–310
Eusebi, V. **54,** 1009–1014
Evan, G. **53,** 1–6
Evan, G. **53,** 713–719
Evan, G.I. **54,** 761–769
Evan, G.I. **53,** 339–344
Evan, G.I. **53,** 331–337
Ewertz, M. **53,** 701–703

### F

Faichney, A. **54,** 1015–1018
Faichney, A. **54,** 587–594
Faivre, J. **53,** 811–815
Farrow, G.M. **53,** 799–804
Fasoli, M. **54,** 311–317
Favrot, M.C. **54,** 637–641
Fetherston, C.A. **53,** 129–135
Fex, G. **53,** 687–690
Fielding, J.W.L. **53,** 203–210
Finan, P.J. **54,** 327–330
Finerty, S. **54,** 385–391
Finlay, I.G. **53,** 231–235
Finney, K.J. **53,** 137–139
Flatt, P.R. **54,** 685–692
Fodstad, O. **53,** 839–841
Fontaniere, B. **54,** 637–641
Forman, D. **53,** 653–659
Formelli, F. **54,** 223–233
Formelli, F. **54,** 33–42
Forskitt, S. **53,** 539–545
Fox, H. **53,** 321–329
Fox, M. **53,** 571–574
Fox, N.E. **53,** 585–594
Fox, N.E. **53,** 529–537
Fozard, J.B.J. **53,** 477–481
Franceschi, S. **54,** 311–317
Franceschi, S. **53,** 817–821
Franko, A.J. **54,** 453–457
Franzen, G. **53,** 643–651
Frappaz, D. **54,** 637–641
Fraumeni, J.F. **54,** 825–832
Freshney, R.I. **54,** 423–429
Freshney, R.I. **53,** 129–135
Frew, A.J. **53,** 555–556
Freyer, J.P. **53,** 345–353
Friedlander, M.L. **53,** 459–464
Frisch, R.E. **54,** 841–845
Fruzynski, I.A. **54,** 669–675
Fukatsu, T. **54,** 699–704

### G

Gaedicke, G. **53,** 181–188
Galante, E. **54,** 833–836
Gallus, G. **54,** 833–836
Galton, D.A.G. **53,** 175–180
Ganapathi, R. **53,** 561–566
Gatter, K.C. **53,** 555–556
Gentet, J.C. **54,** 637–641
Gentile, A. **54,** 311–317
Gentilhomme, O. **54,** 637–641
Geroni, C. **54,** 515–518
Ghosh, A.K. **53,** 555–556
Gibson, D.P. **53,** 265–269
Gibson, F.M. **54,** 771–778
Gilby, E.D. **53,** 539–545
Giles, G.R. **54,** 327–330
Giles, G.R. **53,** 53–57
Giles, G.R. **53,** 477–481
Giles, H.J. **54,** 493–500
Giuliani, F.C. **54,** 515–518
GIVIO **54,** 319–326
Glatstein, E. **53,** 733–736
Glennie, M.J. **54,** 807–818
Godal, A. **53,** 839–841

Goldman, J.M. **53**, 175–180
Gollow, M.M. **54**, 669–675
Gomm, S. **54**, 963–967
Gordon, L.G. **53**, 75–80
Gordon, M. **53**, 761–772
Gorin, N.C. **53**, 737–742
Grabowski, D. **53**, 561–566
Grace, J. **54**, 787–790
Graham, M.A. **53**, 601–606
Grandi, M. **54**, 515–518
Grdina, D.J. **54**, 933–941
Gregory, C.D. **54**, 385–391
Gregory, W.M. **53**, 175–180
Grill, V. **54**, 743–748
Gronstad, K.O. **54**, 251–256
Gugliotta, P. **54**, 631–636
Gullick, W. **54**, 265–269
Gulyas, S. **53**, 743–751
Gupta, S. **54**, 235–238
Gusterson, B.A. **53**, 223–229
Guy, H. **53**, 811–815
Guy, K. **53**, 161–173
Guzzon, A. **54**, 833–836

### H

Haberland, F. **53**, 697–699
Habib, N.A. **54**, 885–889
Habib, N.A. **53**, 697–699
Hadjimichael, O.C. **53**, 281–284
Haensch, C.F. **54**, 431–437
Haglund, C. **54**, 897–901
Haglund, C. **54**, 297–303
Haglund, C. **53**, 189–195
Haglund, C. **53**, 197–202
Halila, H. **54**, 297–303
Hall, E.J. **54**, 919–924
Hall, G.W. **53**, 81–89
Hall, M.J. **53**, 595–600
Hamilson, P.S. **54**, 587–594
Haneda, K. **53**, 557–560
Haney, L. **54**, 459–465
Hansen, H.S. **54**, 7–17
Hansen, H.H. **54**, 7–17
Hansen, M. **54**, 7–17
Hardcastle, J.D. **53**, 37–45
Hardell, L. **54**, 115–117
Harousseau, J.L. **53**, 737–742
Harrington, F. **53**, 733–736
Harris, A.L. **54**, 657–660
Harris, A.L. **53**, 575–578
Harris, A.L. **53**, 361–368
Harris, A.L. **54**, 119–122
Harris, M. **54**, 819–823
Harris, R.W.C. **53**, 653–659
Hart, I.R. **53**, 255–263
Hartley, A.L. **54**, 819–823
Harvey, K. **54**, 587–594
Harvie, R. **53**, 211–215
Harwood, P.J. **54**, 75–82
Harwood, P.J. **54**, 341–344
Hasleton, P.S. **54**, 963–967
Hasleton, P. **54**, 265–269
Hasleton, P.S. **53**, 453–457
Hasleton, P.S. **53**, 285–287
Haug, E. **53**, 839–841
Hawkey, C.J. **54**, 871–875
Hawkins, R.A. **53**, 407–410
Hay, I.D. **53**, 799–804
Hayakawa, T. **53**, 773–777
Healy, K. **54**, 265–269
Heath, L. **54**, 771–778
Heighway, J. **53**, 285–287
Heighway, J. **53**, 453–457
Heilbrun, L.K. **54**, 677–683
Heinerman, E.C.M. **54**, 53–59
Heiremans, J. **54**, 431–437
Hepburn, P.J. **54**, 131–135
Herman, A.G. **54**, 431–437
Herrera, G.A. **53**, 411–414
Hershman, M. **54**, 885–889
Hershman, M.J. **53**, 697–699
Herve, P. **53**, 737–742
Hietel, B. **54**, 239–243
Hill, M.J. **53**, 145–147
Hill, R.P. **53**, 743–751
Hince, T.A. **54**, 557–568
Hirst, D.G. **53**, 247–254
Hoal-Van-Helden, E.G. **54**, 287–295
Hodson, B.A. **54**, 853–855
Hodson, B.A. **54**, 623–629
Holland, J.F. **54**, 415–421
Holme, T.C. **53**, 465–476
Holmes, C.H. **54**, 871–875
Hooker, G. **53**, 727–732
Hoover, R. **54**, 825–832
Horsfall, D.J. **53**, 23–28
Horsman, M.R. **53**, 247–254
Hoshi, K. **53**, 557–560
Houmayoun-Valyani, S.D. **54**, 447–452
Howard, J.K. **54**, 493–500
Howie, S.E.M. **53**, 707–711
Hoyes, A.D. **53**, 81–89
Hug, V.M. **54**, 607–613
Hughes, G. **54**, 119–122
Huhtala, M.L. **54**, 297–303
Humm, J.L. **54**, 863–870

### I

Iliakis, G. **54**, 245–249
Ince, P. **53**, 137–139
Ingleton, P. **54**, 857–859
Ioannou, S. **54**, 717–725
Iqbal, M.J. **54**, 447–452
Iwaguchi, T. **54**, 91–96

### J

Jack, A. **54**, 519–523
Jack, A.S. **53**, 713–719
Jacobson, G. **54**, 377–378
Jalanko, H. **53**, 197–202
James, O.F.W. **53**, 361–368
Jani, K.H. **53**, 141–143
Jeekel, J. **54**, 337–339
Jensen, O.M. **54**, 483–492
Joannou, C. **54**, 791–798
Joher, A. **54**, 401–408
Johnson, B. **54**, 123–126
Johnson, R.T. **54**, 345–376
Johnson, R.J. **53**, 623–628
Johnson, S.A. **53**, 175–180
Jones, D.B. **54**, 277–286
Jones, M. **53**, 793–797
Jori, G. **53**, 615–621
Joseph, A.E.A. **54**, 511–513
Judson, I.R. **53**, 601–606

### K

Kalima, T.V. **54**, 837–840
Kamiya, H. **54**, 711–715
Kanclerz, A. **54**, 693–698
Kato, T. **53**, 369–375
Katz, F. **54**, 771–778
Kaur, H. **54**, 717–725
Kaye, S.B. **54**, 519–523
Kaye, S.B. **54**, 423–429
Kaye, S.B. **54**, 19–23
Kaye, S.B. **53**, 129–135
Keersmaekers, G.M. **54**, 431–437
Kefford, R.F. **54**, 787–790
Kellie, S. **53**, 465–476
Kelsey, K.T. **54**, 107–114
Kemshead, J.T. **54**, 771–778
Kemshead, J.T. **53**, 289–292
Kemshead, J. **54**, 637–641
Kemshead, J.T. **54**, 83–90
Keng, P.C. **54**, 25–32
Kerr, A.M. **54**, 423–429
Kerr, D.J. **54**, 475–482
Kerr, D.J. **54**, 423–429
Kerr, H. **54**, 587–594
Kerr, I.B. **53**, 231–235
Kerr, I.B. **53**, 713–719
Kew, M.C. **54**, 779–785
Kimura, K. **53**, 557–560
Kind, P.R.N. **53**, 761–772
King, D.J. **53**, 695–696
Kingsnorth, A.N. **53**, 683–686
Kingston, J.E. **53**, 661–671
Kirkland, S.C. **53**, 779–785
Kiviranta, H. **53**, 507–512
Klein, M. **53**, 181–188
Klintenberg, C. **54**, 271–276
Klintenberg, C. **53**, 643–651
Klugmann, F.B. **54**, 743–748
Knox, P. **54**, 511–513
Koch, C.J. **54**, 453–457
Koch, G.L.E. **53**, 13–22
Kofina, A. **54**, 651–656
Komada, Y. **54**, 711–715
Komuro, C. **54**, 749–754
Konno, A. **53**, 369–375
Konstantinidou, N. **54**, 651–656
Kopelyan, I. **53**, 823–827
Kottapally, S. **54**, 305–310
Krajewski, A. **53**, 407–410
Krajewski, A.S. **53**, 161–173
Krausz, T. **53**, 727–732
Kuentz, M. **53**, 737–742
Kuppen, P.J.K. **54**, 951–955
Kuusela, P. **53**, 197–202

### L

La-Vecchia, C. **54**, 311–317
La-Vecchia, C. **53**, 817–821
Laekeman, G.M. **54**, 431–437

Lafleur, M.V.M. **53,** 829–833
Lai, C.L. **54,** 67–73
Lamuraglia, G.N. **53,** 683–686
Landoni, F. **54,** 925–932
Landoni, F. **53,** 47–52
Landuyt, W. **54,** 579–586
Lange, A. **53,** 811–815
Larsson, L.G. **53,** 673–681
Laverick, M. **53,** 761–772
Le Caer, F. **54,** 501–504
Leach, S. **54,** 123–126
Leake, R.E. **53,** 7–11
Leandoer, L. **53,** 687–690
Lee, C.S. **54,** 779–785
Lee, F.D. **53,** 231–235
Lee, F.D. **53,** 713–719
Lee, P.N. **54,** 97–105
Leeflang, P. **54,** 951–955
Leivonen, M.K. **54,** 837–840
Lennard, L. **53,** 115–119
Lennox, E.S. **53,** 313–319
Lennox, E.S. **53,** 721–725
Lennox, E.S. **53,** 307–312
Leonard, R.C.F. **53,** 575–578
Levi, J. **53,** 211–215
Liber, H.L. **54,** 107–114
Lieber, M.M. **53,** 799–804
Lillehaug, J.R. **53,** 121–127
Lilleyman, J.S. **53,** 115–119
Lin, G.S. **53,** 265–269
Lin, H.J. **54,** 67–73
Lin, T.M. **54,** 969–976
Lin, T.M. **53,** 399–405
Lindberg-Navier, I. **54,** 377–378
Lindgren, J. **53,** 189–195
Ling, L.L. **54,** 911–917
Little, J.B. **54,** 107–114
Loadman, P.M. **54,** 595–600
Lollini, P.L. **54,** 1009–1014
Lombardero, J. **54,** 271–276
Lorico, A. **54,** 925–932
Losowsky, M.S. **54,** 857–859
Lowe, A.D. **53,** 313–319
Lowe, A.D. **53,** 721–725
Lowe, A.D. **53,** 307–312
Lowe, D. **54,** 705–709
Lowry, W.S. **53,** 75–80
Luk, C.K. **54,** 25–32
Lykkesfeldt, A.E. **53,** 29–35

### M

MacDonald, F. **53,** 203–210
MacFarlane, G.J. **54,** 475–482
Machin, J. **54,** 669–675
Macias, A. **54,** 271–276
Mackillop, W.J. **54,** 661–667
Madda, P. **53,** 497–500
Maddocks, J.L. **53,** 115–119
Maisin, H. **54,** 61–66
Makela, O. **53,** 197–202
Mallardi, F. **54,** 743–748
Malt, R.A. **53,** 683–686
Mangioni, C. **54,** 925–932
Mangioni, C. **53,** 47–52
Mantovani, A. **53,** 47–52
Maraninchi, D. **53,** 737–742
Marcus, R.E. **53,** 175–180
Mariani, M. **54,** 33–42
Maritaz, O. **54,** 637–641
Markowe, H.L.J. **53,** 59–64
Marks, V. **54,** 685–692
Marquet, R.L. **54,** 337–339
Marsden, H.B. **54,** 819–823
Marshall, V.R. **53,** 23–28
Marshall, R.J. **53,** 53–57
Martin, J.A. **53,** 585–594
Martin, P.M. **54,** 501–504
Marty, M. **53,** 737–742
Mason, D.Y. **53,** 555–556
Masters, J.R.W. **54,** 131–135
Matlin, S.A. **54,** 511–513
Matsui, Y. **53,** 415–417
Matsuzawa, A. **54,** 91–96
Matthews, J. **53,** 787–791
McArdle, C.S. **54,** 519–523
McArdle, C.S. **53,** 231–235
McArdle, C.S. **53,** 835–838
McBride, H.M. **54,** 807–818
McBride, W.H. **53,** 707–711
McCarthy, W.H. **54,** 787–790
McCormack, M.A. **53,** 7–11
McDowall, M.E. **53,** 271–279
McElwain, T.J. **53,** 607–614
McKcant, E.L. **53,** 23–28
McMillan, T.J. **53,** 753–759
McMurdo, D.F.S. **54,** 519–523
Meigs, J.W. **53,** 281–284
Melhuish, P.B. **54,** 257–263
Melton, R.G. **53,** 377–384
Merry, S. **53,** 129–135
Milan, C. **53,** 811–815
Millar, J.L. **53,** 607–614
Minden, M.D. **53,** 459–464
Minowada, J. **53,** 181–188
Mircheva, J. **53,** 105–114
Mircheva, J. **53,** 99–103
Mishal, Z. **54,** 501–504
Mitchell, J.B. **53,** 733–736
Moerkerk, P.M. **54,** 409–414
Mogami, H. **53,** 773–777
Moller, T.R. **53,** 579
Moore, M. **54,** 891–895
Morasca, L. **54,** 925–932
Morgan, D.R. **54,** 643–649
Morra, I. **54,** 631–636
Moss, D.W. **53,** 483–487
Mott, M.G. **54,** 385–391
Moyes, P. **54,** 587–594
Moyes, P. **54,** 1015–1018
MRC Working Party **53,** 513–518
Mueller, H.W. **53,** 385–391
Mueller-Klieser, W. **53,** 345–353
Munro, A.J. **54,** 885–889
Munro, A.J. **53,** 727–732
Munro, A.J. **54,** 891–895

### N

Nagasaka, T. **54,** 699–704
Nagy, B. **54,** 933–941
Nakashima, N. **54,** 699–704
Nanni, P. **54,** 1009–1014
Nauta, M.M. **54,** 331–335
Neefe, J.R. **54,** 401–408
Negri, E. **54,** 311–317
Negri, E. **53,** 817–821
Newlands, E.S. **53,** 91–97
Newman, S. **53,** 377–384
Nias, A.H.W. **53,** 761–772
Nicholson, R.I. **54,** 903–909
Nicholson, R.I. **53,** 629–636
Nickers, P.H. **54,** 61–66
Nicol, A. **54,** 1015–1018
Nicoletti, G. **54,** 1009–1014
Nicolson, G.L. **54,** 459–465
Nilsson, O. **54,** 251–256
Nishidai, T. **54,** 749–754
Noel, P. **53,** 637–642
Nohammer, G. **53,** 217–222
Nomura, A. **54,** 677–683
Nordling, S. **53,** 189–195
Nordling, S. **54,** 297–303
Norell, S. **54,** 377–378
Nusse, M. **54,** 245–249

### O

O'Brien, C.J. **54,** 643–649
O'Brien, C. **53,** 393–398
O'Connor, P.J. **53,** 571–574
O'Sullivan, B. **54,** 661–667
Ohnuma, T. **54,** 415–421
Oikawa, T. **54,** 91–96
Okamoto, Y. **53,** 369–375
Okudaira, Y. **53,** 415–417
Olin, R. **54,** 377–378
Olofsson, J. **53,** 643–651
Olsson, H. **53,** 579
Ono, K. **54,** 749–754
Oosters, L. **54,** 61–66
Orell, S.R. **53,** 23–28
Orntoft, T. **53,** 691–694
Osterlind, A. **53,** 501–505
Osterlind, K. **54,** 7–17

### P

Pani, P. **54,** 305–310
Papayannis, A. **54,** 651–656
Papp, L. **53,** 697–699
Paraskeva, C. **54,** 799–805
Parazzini, F. **54,** 311–317
Parmiani, G. **54,** 223–233
Parmiani, G. **54,** 33–42
Parsons, M.A. **54,** 857–859
Patel, S. **54,** 257–263
Paterson, A.C. **54,** 779–785
Paterson, M.E.L. **53,** 705–706
Peacock, J.H. **53,** 237–245
Pearce, N.E. **54,** 493–500
Pectasides, D. **54,** 891–895
Pectasides, D. **53,** 727–732
Pectasides, D. **54,** 885–889
Pedley, R.B. **54,** 341–344
Pedley, R.B. **53,** 377–384
Peltokallio, P. **54,** 837–840
Pepe, S. **54,** 925–932
Peri, G. **53,** 47–52
Perkins, A.C. **53,** 37–45

Peto, J. **54,** 381–383
Philip, I. **54,** 637–641
Philip, T. **53,** 737–742
Philip, T. **54,** 637–641
Phillips, D. **53,** 255–263
Pietribiasi, F. **54,** 631–636
Pimm, M.V. **53,** 37–45
Pincott, J.R. **54,** 83–90
Pinedo, H.M. **54,** 331–335
Pinkerton, R. **53,** 737–742
Piris, J. **54,** 877–883
Pirro, J.P. **54,** 919–924
Pitts, A.M. **53,** 411–414
Plowman, P.M. **54,** 903–909
Pluimackers-Westmijze, E.J. **53,** 829–833
Plumb, J.A. **53,** 129–135
Pollard, R.B. **53,** 567–570
Poulsen, H. **53,** 691–694
Powell, C.J. **54,** 685–692
Powell, M. **54,** 393–400
Pretlow, T.G. **53,** 411–414
Pretlow, T.P. **53,** 411–414
Price, M.R. **54,** 393–400
Prochazka, A.V. **53,** 59–64
Prodi, G. **54,** 1009–1014
Prosperi, E. **54,** 943–950
Prosperi, E. **54,** 33–42

### Q

Quash, G. **53,** 637–642

Quirke, P. **54,** 643–649

Quirke, P. **54,** 327–330

Quirke, P. **53,** 477–481

### R

Rahman, A. **54,** 401–408
Rainwater, L.M. **53,** 799–804
Raleigh, J.A. **54,** 453–457
Ralfkiaer, N. **53,** 555–556
Rampling, R. **53,** 727–732
Ranson, D.L. **54,** 385–391
Ranstam, J. **53,** 579
Rao, K.N. **54,** 305–310
Rawlins, G. **54,** 75–82
Read, G. **53,** 623–628
Reddi, E. **53,** 615–621
Reeve, J.G. **53,** 519–528
Reilly, F. **54,** 705–709
Richards, I.D.G. **53,** 393–398
Richardson, T.C. **54,** 807–818
Richardson, R.B. **53,** 289–292
Richmond, F. **54,** 771–778
Rickinson, A.B. **54,** 385–391
Ringback, G. **53,** 507–512
Risberg, B. **53,** 643–651
Ritson, A. **54,** 761–769
Ritson, A. **53,** 331–337
Ritson, A. **53,** 339–344
Roberts, B. **53,** 393–398
Roberts, C. **54,** 787–790
Roberts, J. **54,** 733–741
Roberts, P.J. **53,** 189–195
Roberts, P.J. **54,** 297–303
Roberts, P.J. **53,** 197–202
Robins, R.A. **53,** 37–45
Robinson, A. **54,** 877–883
Robinson, B.A. **53,** 607–614
Rofstad, E.K. **54,** 569–578
Rogers, G.T. **54,** 341–344
Rogers, G.T. **54,** 75–82
Rorth, M. **54,** 7–17
Ross, C.A. **53,** 7–11
Ross, J.S. **53,** 683–686
Rossi, C. **54,** 223–233
Rossini, S. **53,** 47–52
Rowe, M. **54,** 385–391
Russo, A. **53,** 733–736
Russo, D. **53,** 733–736
Rutty, C.J. **53,** 601–606

### S

Saario, I.A. **54,** 837–840
Saggin, L. **54,** 1009–1014
Sakamoto, Y. **53,** 773–777
Sakurai, M. **54,** 711–715
Sanders, B.M. **53,** 661–671
Sangster, K. **53,** 407–410
Sangster, G. **54,** 19–23
Santini, J. **54,** 755–760
Sany, J. **53,** 805–810
Sarraf, C.E. **54,** 989–998
Satoh, T. **53,** 557–560
Saunders, N. **53,** 705–706
Sawada, M. **53,** 415–417
Schauenstein, E. **53,** 217–222
Scheinin, T.M. **54,** 297–303
Scheinin, T.M. **53,** 197–202
Schiaffino, S. **54,** 1009–1014
Schiff, I. **54,** 841–845
Schluper, H.M.M. **54,** 331–335
Schmidt, H. **53,** 561–566
Schneider, M. **54,** 755–760
Scott, C.A. **53,** 595–600
Searle, F. **53,** 377–384
Seigneurin, J.M. **54,** 501–504
Self, C. **54,** 891–895
Self, C. **54,** 885–889
Serwadda, D. **53,** 497–500
Sewell, H.F. **53,** 695–696
Shaw, H.M. **54,** 787–790
Sheard, C.R. **54,** 705–709
Sheppard, R.A. **54,** 493–500
Sherriff, S. **53,** 705–706
Sherwood, R.F. **53,** 377–384
Sheth, A.R. **53,** 547–554
Sheth, N.A. **53,** 547–554
Shibamoto, Y. **54,** 749–754
Shimizu, K. **53,** 773–777
Shinozuka, H. **54,** 305–310
Shouval, D. **54,** 779–785
Shoval, S. **54,** 847–851
Shrieve, D.C. **54,** 749–754
Siemann, D.W. **54,** 615–622
Sikora, K. **53,** 331–337
Sikora, K. **53,** 1–6
Sikora, K. **54,** 761–769
Sikora, K. **53,** 339–344
Simha, M.M. **53,** 547–554
Simmonds, A.P. **53,** 443–451
Simmonds, A.P. **54,** 587–594
Simmonds, A.P. **54,** 1015–1018
Skolnik, G. **54,** 251–256
Skoog, L. **54,** 271–276
Skovgaard-Powsen, H. **53,** 691–694
Slater, L.M. **54,** 235–238
Slater, T.F. **53,** 217–222
Slavin, B.M. **53,** 761–772
Slee, P.H.T.J. **54,** 951–955
Smith, A.H. **54,** 493–500
Smith, I.E. **54,** 119–122
Smith, I.E. **54,** 657–660
Smith, J.A. **53,** 223–229
Smith, M.E.F. **54,** 643–649
Smith, M.J. **53,** 13–22
Smith, P. **53,** 733–736
Smith, P.G. **54,** 871–875
Smith, P.J. **53,** 99–103
Smith, P.J. **53,** 105–114
Smyth, J.F. **53,** 355–360
Snook, D. **53,** 727–732
Snow, G.B. **54,** 53–59
Sobue, M. **54,** 699–704
Soukop, M. **54,** 519–523
Soukop, M. **53,** 575–578
Southall, P. **54,** 75–82
Spandidos, D.A. **54,** 877–883
Spandidos, D.A. **53,** 231–235
Sparrow, S. **53,** 793–797
Speakman, H. **54,** 999–1008
Spellman, C.W. **54,** 505–509
Spitzer, G. **54,** 607–613
Stace, B. **53,** 793–797
Stamford, I.F. **54,** 257–263
Stapleton, P.J. **53,** 65–74
Steel, G.G. **53,** 753–759
Steele, D. **54,** 733–741
Stefanoudaki, K. **54,** 651–656
Stemmermann, G.N. **54,** 677–683
Stenman, U.H. **54,** 297–303
Stephens, T.C. **53,** 237–245
Stephens, T.C. **53,** 753–759
Sternby, N.H. **53,** 687–690
Stewart, J. **53,** 1–6
Stewart, J. **53,** 519–528
Stewart, J. **53,** 339–344
Stewart, J. **53,** 331–337
Stewart, M.M. **54,** 787–790
Stewart, S. **53,** 727–732
Storer, A.M. **54,** 475–482
Storm, H.H. **54,** 483–492
Stratford, I.J. **53,** 339–344
Stuart-Harris, R. **54,** 657–660
Stupecky, M. **54,** 235–238
Sunter, J.P. **53,** 137–139
Supino, R. **54,** 223–233
Supino, R. **54,** 33–42
Supino, R. **54,** 943–950
Sutherland, R.M. **54,** 911–917
Sutherland, R.M. **54,** 25–32
Sutherland, C. **54,** 787–790
Sutherland, R.M. **53,** 345–353
Suzuki, F. **53,** 567–570
Svensen, R. **53,** 255–263
Swanston-Flatt, S.K. **54,** 685–692
Sweet, P. **54,** 235–238

### T

Takahashi, M. **54,** 749–754
Takeuchi, J. **54,** 699–704
Talamini, R. **53,** 817–821
Talavera, J.G. **53,** 175–180
Talbot, I.C. **54,** 791–798
Tamakawa, Y. **53,** 369–375
Tan, K.S. **54,** 685–692
Tannert, S. **53,** 385–391
Tannock, I.F. **54,** 733–741
Tannock, I.F. **53,** 823–827
Taylor, C. **54,** 123–126
Taylor-Papadimitriou, J. **53,** 727–732
Teague, C.A. **54,** 493–500
Tee, D.E.H. **54,** 705–709
Teillet, F. **53,** 737–742
Thatcher, N. **54,** 963–967
Thatcher, N. **54,** 265–269
Thatcher, N. **53,** 453–457
Thomas, G.J. **53,** 595–600
Thompson, W.D. **53,** 695–696
Thyss, A. **54,** 755–760
Tilley, W.D. **53,** 23–28
Tisdale, M.J. **54,** 601–606
Tisell, L.E. **54,** 251–256
Tochner, Z. **53,** 733–736
Togawa, K. **53,** 369–375
Togawa, T. **53,** 557–560
Tognoni, G. **54,** 311–317
Toida, M. **54,** 699–704
Tokuda, S. **54,** 505–509
Tomasovic, B. **54,** 607–613
Tomino, L. **53,** 615–621
Tralau, C.J. **54,** 43–52
Traynor, O.J. **53,** 483–487
Treleaven, J. **54,** 771–778
Trivedi, A.H. **53,** 141–143
Tsu, W.T. **54,** 969–976
Tsukidate, K. **54,** 699–704
Tsutui, K. **54,** 749–754
Tubiana, N. **54,** 501–504
Tuominen, L. **54,** 837–840
Turinic, R. **53,** 561–566
Turkes, A. **53,** 629–636
Twentyman, P.R. **53,** 529–537
Twentyman, P.R. **53,** 585–594
Twentyman, P.R. **53,** 519–528

### U

Ubezio, P. **54,** 925–932
Ugelstad, J. **54,** 771–778
Underwood, J.C.E. **54,** 857–859
Urtasun, R.C. **54,** 453–457
Ushio, Y. **53,** 773–777
Uyttenbroeck, F.L. **54,** 431–437

### V

Vagero, D. **53,** 507–512
Valenzyela, R. **53,** 561–566
Vallicioni, J. **54,** 755–760
Van-Den-Berg, L. **54,** 951–955
Van-Den-Berghe, J.A. **54,** 83–90
Van-Der-Schueren, E. **54,** 579–586
Van-Oosterom, A.T. **54,** 951–955
Vergani, D. **54,** 705–709
Vergote, I.B. **54,** 431–437
Verstijnen, C. **54,** 409–414
Vessey, M.P. **53,** 653–659
Vigar, E. **54,** 123–126
Vlachos, J. **54,** 651–656
Vowden, P. **53,** 313–319
Vowden, P. **53,** 721–725
Vowden, P. **53,** 307–312
Vyas, R.C. **53,** 141–143

### W

Wald, N. **54,** 957–961
Wald, N.J. **53,** 653–659
Walker, A.R.P. **53,** 489–495
Walker, A.J. **53,** 489–495
Walker, B.F. **53,** 489–495
Walker, K.J. **54,** 903–909
Walker, K.J. **53,** 629–636
Walker, R. **54,** 459–465
Wallington, T.B. **53,** 289–292
Wamukota, W. **53,** 497–500
Ward, G.K. **54,** 661–667
Waters, E.D. **54,** 669–675
Watson, A.J. **53,** 137–139
Watson, J.V. **53,** 519–528
Watson, J.V. **53,** 331–337
Watson, J.V. **54,** 761–769
Watson, J.V. **53,** 1–6
Weimar, W. **54,** 337–339
Westmacott, D. **53,** 595–600
Wetzel, M.W. **54,** 235–238
Wheldon, T.E. **54,** 423–429
Whitaker, K.B. **53,** 483–487
White, R.D. **54,** 717–725
Whitmore, G.F. **53,** 743–751
Wiechel, K.L. **54,** 377–378
Wieman, T.J. **54,** 43–52
Wiggers, T. **54,** 409–414
Wigley, C.B. **54,** 799–805
Wilkinson, P.M. **53,** 623–628
Wilkinson, P.M. **54,** 891–895
Williams, W.L. **54,** 505–509
Williams, C.M. **54,** 439–446
Williams, A.R.W. **54,** 877–883
Williams, M.R. **53,** 629–636
Williamson, J.M.S. **54,** 643–649
Williamson, C. **53,** 65–74
Williamson, R.C.N. **53,** 697–699
Willox, J.C. **54,** 19–23
Wilson, E.L. **54,** 287–295
Winfield, U.J. **54,** 717–725
Wood, C.B. **53,** 483–487
Wood, C.B. **54,** 885–889
Wood, C.B. **53,** 697–699
Woodruff, M.F.A. **54,** 623–629
Woodruff, M.F.A. **54,** 853–855
Woolley, D.E. **54,** 459–465
Workman, P. **53,** 585–594
Wraight, P. **54,** 761–769
Wright, D.H. **54,** 277–286
Wright, K.A. **53,** 529–537
Wright, K.A. **53,** 585–594
Wu, H.Y. **53,** 399–405
Wu, P.C. **54,** 67–73
Wulfrank, D. **53,** 519–528
Wyke, J.A. **53,** 465–476
Wyllie, A.H. **54,** 877–883
Wyshak, G. **54,** 841–845

### Y

Yano, E. **54,** 107–114
Yen, A. **53,** 561–566
Yonemoto, H. **53,** 557–560
Yoshida, T. **53,** 773–777
You, S.L. **53,** 399–405
Young, L.F. **53,** 843–844

### Z

Zahoor, A. **53,** 829–833
Zalcberg, J.R. **53,** 459–464
Zanaboni, F. **53,** 47–52
Zucker, J.M. **54,** 637–641